# Supplementary material for: Learning curve and surgical outcome of robotic assisted colorectal surgery with ERAS program
Source: Sci Rep. 2022 Nov 29;12:20566. doi: 10.1038/s41598-022-24665-w (PMC9709162; doi:10.1038/s41598-022-24665-w)
Supplement: Supplementary file 2 — Supplementary Information 2. [file 41598_2022_24665_MOESM2_ESM.zip › RAL Raw data-Ñ[▒K/110/Patho_ñw╝╨░O▒Kñσ.pdf]

臺中榮民總醫院 臺中市臺灣大道四段1650號 病理部活體組織切片 臨床醫學實驗及病理診斷中心 檢驗(查)報告

[redacted]

Pathologic diagnosis:
1. Rectum, robotic low anterior resection --- Status post-neoadjuvant radiotherapy with no residual invasive tumor. Ulceration and focal mild mucosal dysplasia are noted. The bilateral cut ends and perirectal lymph nodes (0/12) are free of tumor.
2. Rectum, cut end, distal, resection --- Chronic inflammation.
3. IMA root, dissection --- Unremarkable fibrovasculoadipose tissue.

Prognostic and predictive factor:
1. Tumor size: No residual tumor found.
2. Depth of invasion: No residual tumor found.
3. Histologic grade: Not applicable.
4. Angiolymphatic invasion: Not identified.
5. Perineural invasion: Not identified.
6. Tumor deposits (discontinuous extramural extension): Not identified.
7. Macroscopic tumor perforation: Not identified.
8. Surgical Margins: a) Proximal Margin: Uninvolved by invasive carcinoma. b) Distal Margin: Uninvolved by invasive carcinoma. c) Radial or Mesenteric Margin: Uninvolved by invasive carcinoma.
9. Lymph node status: Uninvolved (0/12).
10. Extranodal involvement: Not identified.
11. Tumor regression grading S/P CCRT: Present, no viable cancer cells (complete response, score 0) (neoadjuvant radiotherapy in this case).
12. Type of polyp in which invasive carcinoma arose: Not applicable.
13. Additional pathologic findings: Not identified.
14. Pathological TNM Stage: ypT0N0(According to the eighth edition, American Joint Committee on Cancer Staging Guidelines for Tumors).
15. TNM descriptors: y (Post-treatment).

Gross description:
The specimen consists of 1) a segment of colon, 13 cm in length and 4.5 cm in proximal cut end circumference, 6.5 cm in distal circumference, and perirectal soft tissue. A gray tan firm annular elevated and centrally ulcerative lesion, 1.5x1 cm, is noted, 0.8 cm to the distal cut end. On cross section, the lesion confined to the mucosa with 1.5 cm to the radial margin. The both cut ends are free of lesion grossly. 2) distal cut end, a piece of tan mucosa-covered soft tissue, 2x0.5x1 cm. 2) IMA root, a piece of tan yellow soft tissue, 1.5x0.5x0.2 cm. Representative part for section: A) proximal cut end B-F) tumor with distal cut end G) specimen 2 H) all of specimen 3 J-K) mesocolic lymph nodes.
#T-59600_2 #M-09413_2 0 1105 00SP00
#T-C4560_2 #M-09183_2 0 1105 000000

已電子簽章

敬告：本單僅供醫師參考使用，不具任何申請效力！！
請病患妥為保管，若不慎遺失需自負個人資料外洩責任！
